# Complications, Implications, and Prevention of Electrosurgical Injuries: Corner Stone of Diathermy Use for Junior Surgical Trainees

**DOI:** 10.1055/s-0037-1606547

**Published:** 2017-09-14

**Authors:** Manjunath Siddaiah-Subramanya, Kor Woi Tiang, Masimba Nyandowe

**Affiliations:** 1 Department of Surgery, Logan Hospital, Meadowbrook, Queensland, Australia; 2 Department of Surgery, Townsville Hospital, Townsville, Queensland, Australia

**Keywords:** diathermy, electrosurgery, prevention, surgical trainees

## Abstract

Diathermy is commonly used in modern-day surgery. The incidence of electrosurgical injuries related to diathermy is under reported, as it is difficult to ascertain the true impact on both patient and healthcare professionals. As junior surgical trainees, understanding of the mechanism and biophysics of the electrosurgical tools enables safer usage and contributes to improved outcome. Careful use of electrosurgical tools during operation and appropriate communication amongst staff members are pivotal to a safe surgical outcome. Here, we discuss the causes and risk factors regarding electrosurgical complications along with suggestions to ensure safe practice, focusing on commonly neglected areas.

Since its invention by Bovie and first use by Cushing in the removal of intracranial tumors in 1927, electrosurgery is now an integral part of modern surgery. 1 Injuries and equipment loss resulting from diathermy use are much more common than what we might think. 2 The true incidence is probably under reported and also difficult to determine. It is currently estimated that around 500 to 600 surgical fires occur annually in the United States. 3 In spite of this, not all the surgical trainees or even consultants have a good understanding of electrosurgery. 4 Approximately, two-thirds of the injuries may not be detected during the procedure. 5 Unfortunately, this has not only implications on the patient care but also immensely increases the financial burden on the health institutes costing them millions of dollars every year. 3

## Biophysics of Diathermy and Its Effects on Cells

To understand the etiology and pathophysiology behind electrosurgery, it is initially important to understand the working of an electrosurgical unit and its different components. This will aid us to effectively manage and more importantly prevent electrosurgical injuries. An electrosurgical circuit includes electrosurgical unit (ESU), active electrode, patient, and the dispersive electrode. The ESU receives alternative current (AC) from the wall supply (50–60 Hz) and converts it into electromagnetic current in excess of 200 kilohertz (KHz). 6 Most modern ESUs are capable of producing current up to 850 KHz 7 that is transferred to the patient via an active electrode (handheld device). The dispersive electrode (diathermy plate) then carries the current from the patient back to ESU, thereby forming an isolated circuit, which is the present-day model without requiring any grounding. This is in contrast to the initial models, which were ground-referenced circuits. Thus, the radiofrequency electrosurgery is always bipolar, it is only the active electrode that is either monopolar or bipolar. An active electrode does not develop heat by itself in contrary to what many believe. The heat is produced in the cells and tissues, which is then transferred back to the electrode that makes it hot. The dispersive electrode must be at least 70 cm ^2^ in area to carry sufficient current back to ESU to avoid inadvertent thermal injury at the site. The dispersive electrode has two electrodes on a single plate, each individually acting as a sensor in case of nonuniform application of the pad. There is a sequential conversion of electromagnetic energy to kinetic energy to thermal energy within the cells and tissues. This whole process is referred to as and actually is “diathermy.” 8 Cauterization on the other hand is a mere transfer of heat from one conducting source to another.

The ESU has primarily two different outputs or waveforms. These include low-voltage continuous output and high-voltage interrupted output. These two outputs bring about vaporization, and desiccation and coagulation of the tissues. The low voltage is commonly referred to and many a times labeled on the ESU as “cut” mode (yellow button or pad on ESU) and is a misnomer. Similarly, high-voltage output is commonly labeled as “coagulation” mode (blue button or pad) that is also a misnomer. There are various other settings to modulate these two outputs as needed. Some of the common modulations include “cut,” “blend,” “desiccate and coagulate,” and “fulgurate or spray.” Cut is linear vaporization caused by moving the electrode in a linear or curvilinear manner. Blend is modulating the duty cycle on low-voltage output. Duty cycle is the percentage of time occupied by the waveform or output. In low-voltage continuous waveform, the duty cycle is 100%. By modulating the duty cycle, voltage could be slightly increased or decreased. 9 In any given ESU, there are levels, whereby one can set a duty cycle. The higher the level (numerically, e.g., level 3 compared with level 1), shorter the duty cycle and higher the voltage. The low-voltage output irrespective of modulation is 600 to 700 V producing temperatures up to 1000°C. In contrary, high-voltage output, which has a duty cycle of approximately 6%, is 3 to 8 kV producing low temperatures. 10

The energy generated within the cells result, essentially, in two types of reaction depending on the temperature that the energy achieves. Tissue reaction varies at different frequencies. At a high frequency of more than 100 KHz, the muscles and nerves do not depolarize. Desiccation refers to efflux of water molecules out of the cells resulting in their shrinkage. Coagulation refers to protein denaturation. Desiccation and coagulation occur simultaneously at 60 to 90°C. Vaporization occurs above 100°C and refers to conversion of water to gas resulting in rapid expansion of cell volume and rupture of cell membrane and wall. Both vaporization and desiccation and coagulation are results of application of low-voltage continuous current. The difference lies in the manner the active electrode is applied to the tissues. If the electrode is used in a noncontact manner close to the tissues, it results in vaporization due to development of higher temperatures. On the other hand, if the electrode is used in a contact manner with the tissue, it results in desiccation and coagulation.

Fulguration refers to superficial coagulation over a wider surface area. It is also known as spray mode. Here, the active electrode is used in a noncontact manner and is a result of high-voltage interrupted current with a very low duty cycle. In practice, this is the mode employed to desiccate and coagulate tissues in general using a monopolar electrode; the electrode is used in a contact manner. The reason is that it leads to superficial desiccation over a wider area that is often sufficient for nonvascular tissues or very small blood vessels. To produce a uniform seal in a blood vessel, low voltage must be used, which unlike high-voltage waveform, coagulates the vessel slowly and produces deeper desiccation with higher temperatures. This effect is observed well with the use of bipolar active electrode or direct coupling technique.

## Complications of Electro Surgery

There exist a variety of injuries caused by electrosurgery. Various authors and guidelines group them in different ways. We present here a more practical approach and have grouped all the possible complications into four sections: thermal-, smoke-, fire-, and explosion-related injuries. In addition, patients with implantable electromagnetic devices need a special mention due to the fact that these patients are increasing in number, and the electrosurgical devices interact differently and cause specific injuries in the presence of these devices.

As with any mechanical or electrical device, when an issue arises in relation to the equipment, there are various approaches one could apply to solve it, especially if the equipment comprises of various elements. However, it is crucial to have a strong foundation and understanding of not only the biophysics of the equipment but also the pathophysiology and mechanism of the injury or adverse event. Henceforth, we have detailed not just the injury related to electrosurgery here, but also the mechanism of injury along with an approach to solve both the issue and prevent the injury ( Table 1 ).

**Table 1:** Mechanism and solutions to various electrosurgery-related injuries

Injury	Mechanism and physics	Solution/Preventative strategies
Thermal	From active electrode	
	Inappropriate contact (too much power or using wrong setting) with target or neighboring tissue leading to direct injury	Correct usage of electrode with recommended/proper settings. Use the lowest possible power (W) Use audible activation to avoid unnecessary prolonged contact with tissues or requesting power to be increased Complete the communication loop between the surgeon and the scout nurse changing the settings
	Inadvertent activation of the active electrode Plastic sleeves may melt and cause thermal damage to underlying tissue deep to the drapes Wetness in the diathermy holder may increase resistance and cause diversion of current	Avoid accidental stepping on the foot pedal (in laparoscopic surgery) Remove electrode from within patient's body when not using (in laparoscopic surgery) Use rigid plastic holder (not plastic sleeves) for active electrode, thereby ensuring it is not wet and there are no other instruments in the holder that may also contribute to injury from diversion of current
	Inappropriate placement of hot electrode on the drapes leading to transmission of heat in patient's skin	Place active electrodes in the plastic holder
	Lateral extension of current on wet skin prepared with skin preparation containing alcohol	Wait for skin preparation to dry before using diathermy
	Vertical extension of current along a vascular pedicle or adhesions	Relax on the pedicle prior to activating the electrode Use of shorter periods of activation (1–2 s)
	From dispersive electrode	
	Loss of contact of the electrode with the patient	Ensure uniform contact of the electrode (both the split electrodes) Ensure skin is clean, dry, and shaved Replace the electrode with a new one if it needs repositioning
	Inappropriate application of electrode causing diversion of current	Avoid bony prominences Do not place adjacent to ECG leads
	From diversion of current	
	Insulation failure leading to dispersion of high-density current. Smaller the break in insulation, higher the density of current dispersed	Regular check of instruments and their insulation by industry engineers using active electrode monitoring systems
	Direct coupling injury: Direct coupling is transfer of current from one conductive source to another. Type of indirect injury: Injury could be to the patient or the surgeon. Inadvertent transfer of current through small breaks that may happen in gloves, particularly an issue for surgeons wearing single glove. The gloves and hand in addition increase impedance encouraging lateral flow of current (15% of new and 50% of used gloves have holes) Instrument in contact with the active electrode may not be completely in view (in laparoscopic surgery) and in contact with a nontarget tissue Increased risk in SILS due to close proximity of instruments	Avoid unnecessary contact of the active electrode with another conductive source (e.g., retractors) When using direct coupling intentionally (as with sealing a blood vessel), ensure that the conductive source is not in contact with a nontarget tissue or another conductive source. Make sure that the active electrode is brought into contact with the conductive source (as in the case of forceps) below the level of the handgrip Surgeons need to wear double gloves Tip of the electrode and the conductive source must always be in view Extra care need to be taken in SILS while crossing the instruments and ensuring that the working segment of all the active instruments are always in view
	Capacitive coupling injury: Capacitive coupling is transfer of current along the length of the active electrode by means of displacement (e.g., when the insulator becomes dielectric), most commonly in laparoscopic surgery Factors increasing the risk: High voltage (coagulation mode), open activation (electrode not in contact with tissue), and factors that increase impedance (wrapping of cable around a metal clamp, such as towel clip and activation over previously coagulated tissue)	Use the lowest power setting possible Activate electrode only after making contact with tissue Avoid factors that increase impedance to flow of current
	Alternate site injury when patient is in contact with another high conductance element such as metal (rare nowadays with isolated generators)	Ensure that patient is not in contact with any metal that he is not supposed to be Extra care need to be taken while using fixed metal retraction system to ensure that there is no contact of the active electrode with any part of that retractor
Smoke	Noxious gases irritating the respiratory tract	Use smoke evacuators and suction
	Carcinogenic elements potentially increasing the risk of cancers	Use smoke evacuators and suction
Fire	Inappropriate use of diathermy near an oxygen source or alcohol skin preparation. This could even cause an explosion	Avoid using diathermy near oxygen source Allow alcohol-based skin preparations to dry Stop the flow of all airway gases to the patients, may need temporal removal of the airway itself Remove the drapes and burnt material from the patient Extinguish fire In extreme situations, follow local fire evacuation protocol
	Inadvertent activation of electrode placed over the drapes or careless placement of hot electrodes over the drapes (exacerbated by nearby oxygen source) resulting drapes catching fire	Place active electrodes in the plastic holder
Explosion	Inappropriate use of active electrode in the vicinity of inflammable gas under pressure (e.g., opening an obstructed colon containing methane)	Use of alternative cutting/dissecting instrument (e.g., scissors)
PPM, ICD and other pacemakers (e.g., sacral nerve stimulator, spinal stimulators)	Interference form electromagnetic current. Rare with modern devices Possible effects include reprograming, damage to PPM or ICD, inhibition, reversion to back up mode and myocardial thermal injury	Use bipolar electrode or advanced bipolar devices when possible When using monopolar electrode, use in short burst for the shortest time period possible Apply dispersive electrode as far as possible from PPM, ICD, and other pacemakers Monitor PPM for inhibition with ECG. Reprogram postoperatively if there is inhibition during surgery or if any major adverse events occur during surgery Deactivate ICD immediately preoperatively and active postoperatively. If deactivation could not be organized with the cardiologist, then a magnet could be placed over the ICD for the entire period of operation In case of cardiac arrest, follow standard ALS guidelines

Abbreviations: ALS, advanced life support; ECG, electrocardiogram; ICD, implantable cardioverter defibrillator; PPM, permanent pacemaker; SILS, single-incision laparoscopic surgery.

## Discussion

Most of the injuries caused by electrosurgery are related to thermal energy and so commonly referred to as thermal injuries. There are other types of injuries with electrosurgery as mentioned above. Thermal injuries can be classified as direct and indirect injuries. Direct injuries are those that are caused by inadvertent use of active electrode on any part of the body apart from the intended organ or tissue. Indirect injuries are those that occur as a result of contact of the tip or any part of the active electrode with any other metal instrument, which in turn, is in contact with the tissue or that injury that occurs out of the operating field due to spread of current from the shaft of the active electrode to nearby tissue.

Smoke-related injuries are commonly due to impaired vision and almost exclusively seen in laparoscopic surgeries. Impaired vision leads to inappropriate contact of the active electrode with tissue, thereby causing thermal injury. The smoke released upon contact of active electrode with tissue contains various noxious elements. 11 These include gases, such as benzene, nitriles, hydrocyanide and other hydrocarbons, and formaldehyde. 12 Some of these are potentially carcinogenic. The smoke also contains bodily products, such as blood, fatty acids, and viruses, which could transmit infections. The thermal energy also converts part of the blood to methemoglobin and carboxyhemoglobins.

Although there are no documented cases of cancers directly related to exposure of smoke produced from diathermy, there have been several cases of minor respiratory illness and ocular irritation. 13 14 These particles could easily be inhaled in spite of wearing masks. The average pore size of a surgical mask is 5 µm, whereas most of the particles contained in the smoke measure between 1.0 and 1.5 µm. 15 It is a current recommendation, although not mandatory, by various work place health and safety organizations in many countries to use suction devices in operating theatres to evacuate such smoke.

Fires in operating theaters are rare but devastating when occur. There are approximately 100 fires cases reported in the United States every year; 85% of them are minor and result in minimal or no injuries with only a few disabling or disfiguring injuries. 16 More than 50% of these injuries occur in the head and neck region. A major contributor is the oxygen supply around the oxygen mask, endotracheal tube, or laryngeal mask airway. 17 18 Explosion-related injuries are rarely seen and if so, are again, most often related to head and neck surgeries. 19 The confined space along with inflammable gas contributes to more severe burns. In the general surgical setting, performing an enterotomy on a dilated colon poses increased risk of explosion, and alternative methods such as scissors are safer. 20

Electromagnetic devices, such as permanent pacemaker, implantable defibrillator, and pacing devices, such as spinal stimulator and sacral nerve stimulators, are increasingly used and tend to interfere with electrosurgical devices. 21 22 Although these are rare with modern electromagnetic devices, they may not be able to be assessed, especially during an emergency operation. The interference caused by diathermy could lead to arrhythmias and potentially be life threatening. 22 A safer approach would be to utilize bipolar electrosurgical methods. Alternatively, it is safe to deactivate the device temporarily before the operation for the entire duration of the operation or to use a magnet intraoperatively. Care must be taken to activate and get the device checked by cardiologist or an industry representative accordingly.

Laparoscopic cholecystectomy and appendectomy are some of the common general surgical operations performed by the junior surgical trainees. Even though the complication rates related to electrosurgery in these operations are low, these could be significant if occur. 23 24 Injuries related to diathermy may not be identified intraoperatively. The injuries often present late with strictures of the bile duct or peritonitis from bile leak or bowel perforation. When using diathermy near any of these structures, a safer technique is to keep away from bile duct and bowel and stimulate the active electrode only for a short period of time to avoid pedicle effect and serosal injury. 2 25

With increasing expertise in endoscopy, therapeutic procedures, such as polypectomy and endoscopic mucosal resections, are becoming more common. Surgical trainees are increasingly getting exposed and trained in these procedures. Diathermy forms a vital part of the practice to achieve hemostasis and maintain a clear filed while performing these procedures. Injury in colon is more likely to happen on the right side, which is thinner. 26 The rates are low at 0.1 to 0.2% and are often recognized only 24 hours postoperatively. Majority of them could be managed nonoperatively with good outcomes. 27 28 On the other hand, endoscopic mucosal resection utilized for excision of early gastrointestinal cancers has a slightly higher rate of perforation at 4 to 6% and may need operative measures to address the perforation. 29 30

Irrespective of the procedure, a thorough knowledge of the electrosurgical unit and patient forms the basis of prevention of diathermy-related injuries for any surgeon. Having a framework to set the patient up and the related necessary equipment before the start of the procedure in such a way that there is minimal interference of the diathermy with the patient are vital. There is no substitute to using the diathermy appropriately and safely.

## Conclusion

There are varied complications that may be encountered with the use of electromagnetic current. Thankfully, almost all of them are preventable and the key lies in taking those preventative measures and following appropriate, safe surgical techniques at every step of the operation. The operator should always exercise caution and good communication to improve situational awareness, ultimately improving the surgical outcome.

## References

[1] Cushing H Meningiomas arising from the olfactory groove and their removal by the aid of electro surgery Lancet 1927 1 1329 1339

[2] Humes D J Ahmed I Lobo D N The pedicle effect and direct coupling: delayed thermal injuries to the bile duct after laparoscopic cholecystectomy Arch Surg 2010 145 01 96 98 20083761 10.1001/archsurg.2009.236

[3] Choudhry A J Haddad N N Khasawneh M A Cullinane D C Zielinski M D Surgical fires and operative burns: lessons learned from a 33-year review of medical litigation Am J Surg 2017 213 03 558 564 28093118 10.1016/j.amjsurg.2016.12.006

[4] McQuail P M McCartney B S Baker J F Kenny P Diathermy awareness among surgeons-an analysis in Ireland Ann Med Surg (Lond) 2016 12 54 59 27895908 10.1016/j.amsu.2016.10.006 PMC5121146

[5] Perantinides P G Tsarouhas A P Katzman V S The medicolegal risks of thermal injury during laparoscopic monopolar electrosurgery J Healthc Risk Manag 1998 18 01 47 55 10176550 10.1002/jhrm.5600180107

[6] Australian 3-pin electrical mains plug. Available at:http://accesscomms.com.au/australian-mains-plug/. Accessed April 29, 2017

[7] Beamer® CE600 System. Endoscopic Electrosurgical Platform.http://www.conmed.com/en/medical-specialties/gastroenterology-and-pulmonology/polypectomy/generators/beamer-ce600-system-endoscopic-electrosurgical-platform2017. Accessed April 4, 2017

[8] Watson A B Loughman J The surgical diathermy: principles of operation and safe use Anaesth Intensive Care 1978 6 04 310 321 736252 10.1177/0310057X7800600404

[9] Soderstrom R M Levy B S Engel T Reducing bipolar sterilization failures Obstet Gynecol 1989 74 01 60 63 2733944

[10] Massarweh N N Cosgriff N Slakey D P Electrosurgery: history, principles, and current and future uses J Am Coll Surg 2006 202 03 520 530 16500257 10.1016/j.jamcollsurg.2005.11.017

[11] Hensman C Baty D Willis R G Cuschieri A Chemical composition of smoke produced by high-frequency electrosurgery in a closed gaseous environment. An in vitro study Surg Endosc 1998 12 08 1017 1019 9685533 10.1007/s004649900771

[12] Moot A R Ledingham K M Wilson P F Composition of volatile organic compounds in diathermy plume as detected by selected ion flow tube mass spectrometry ANZ J Surg 2007 77(1–2):20 23 17295814 10.1111/j.1445-2197.2006.03827.x

[13] Lindsey C Hutchinson M Mellor G The nature and hazards of diathermy plumes: a review AORN J 2015 101 04 428 442 25835008 10.1016/j.aorn.2015.01.021

[14] Dobrogowski M Wesolowski W Kucharska M Health risk to medical personnel of surgical smoke produced during laparoscopic surgery Int J Occup Med Environ Health 2015 28 05 831 840 26224495 10.13075/ijomeh.1896.00374

[15] Pillinger S H Delbridge L Lewis D R Randomized clinical trial of suction versus standard clearance of the diathermy plume Br J Surg 2003 90 09 1068 1071 12945072 10.1002/bjs.4214

[16] Haith L R Jr Santavasi W Shapiro T K Burn center management of operating room fire injuries J Burn Care Res 2012 33 05 649 653 22878495 10.1097/BCR.0b013e31825d6aad

[17] Daane S P Toth B A Fire in the operating room: principles and prevention Plast Reconstr Surg 2005 115 05 73e 75e 10.1097/01.prs.0000157015.82342.21 15809576

[18] Gorphe P Sarfati B Janot F Airway fire during tracheostomy Eur Ann Otorhinolaryngol Head Neck Dis 2014 131 03 197 199 24703002 10.1016/j.anorl.2013.07.001

[19] Partanen E Koljonen V Salonen A Bäck L J Vuola J A patient with intraoral fire during tonsillectomy J Craniofac Surg 2014 25 05 1822 1824 25119404 10.1097/SCS.0000000000001036

[20] Webb J B Balaratnam S Park A J Flame bums: a forgotten danger of diathermy? Surgeon 2003 1 02 111 113 15573632 10.1016/s1479-666x(03)80127-0

[21] Meyer J P Crew J The risks of diathermy in the urological patient with a pacemaker or an automatic internal cardiac defibrillator BJU Int 2008 101 05 528 529 18257854 10.1111/j.1464-410X.2007.07441.x

[22] Gunaruwan P Barlow M Diathermy-induced ventricular fibrillation with Riata high-voltage lead insulation failure Europace 2013 15 04 473 22872605 10.1093/europace/eus227

[23] Taylor A M Li M K Laparoscopic management of complications following laparoscopic cholecystectomy Aust N Z J Surg 1994 64 12 827 829 7980255 10.1111/j.1445-2197.1994.tb04557.x

[24] Slater K Strong R W Wall D R Lynch S V Iatrogenic bile duct injury: the scourge of laparoscopic cholecystectomy ANZ J Surg 2002 72 02 83 88 12074081 10.1046/j.1445-2197.2002.02315.x

[25] Kivnick S Kanter M H Bowel injury from rollerball ablation of the endometrium Obstet Gynecol 1992 79(5, Pt 2):833 835 1565382

[26] Aras A Oran E Seyit H Karabulut M Gök İ Aliş H Colonoscopic perforations, what is our experience in a training hospital? Surg Laparosc Endosc Percutan Tech 2016 26 01 44 48 26679682 10.1097/SLE.0000000000000220

[27] Sagawa T Kakizaki S Iizuka H Analysis of colonoscopic perforations at a local clinic and a tertiary hospital World J Gastroenterol 2012 18 35 4898 4904 23002362 10.3748/wjg.v18.i35.4898 PMC3447272

[28] Iqbal C W Cullinane D C Schiller H J Sawyer M D Zietlow S P Farley D R Surgical management and outcomes of 165 colonoscopic perforations from a single institution Arch Surg 2008 143 07 701 706; discussion, 706–707 18645114 10.1001/archsurg.143.7.701

[29] Yu C Liao G Fan C Long-term outcomes of endoscopic resection of gastric GISTs Surg Endosc 2017 10.1007/s00464-017-5557-2 28424911

[30] Schlottmann F Patti M G Shaheen N J Endoscopic treatment of high-grade dysplasia and early esophageal cancer World J Surg 2017 41 07 1705 1711 28275832 10.1007/s00268-017-3977-8

